# Gemifloxacin resistance in *Mycobacterium tuberculosis* without QRDR mutations in *gyrA* or *gyrB*: evidence for non-canonical resistance mechanisms

**DOI:** 10.1128/spectrum.03710-25

**Published:** 2026-06-15

**Authors:** Niloofar Hekmatpour, Mina Hajizadeh, Shirin Yousefi, Majid Marjani, Samira Tarashi, Seyed Davar Siadat

**Affiliations:** 1 Department of Microbiology, Faculty of Advanced Science and Technology, Tehran Medical Sciences, Islamic Azad University 680680, Tehran, Iran; 2 Advanced-Sciences and Technology Faculty, Islamic Azad University, Tehran Medical Branch 68106 , Tehran, Iran; 3 Clinical Tuberculosis and Epidemiology Research Center, National Research Institute of Tuberculosis and Lung Diseases (NRITLD), Shahid Beheshti University of Medical Sciences 556492, Tehran, Iran; 4 Department of Mycobacteriology and Pulmonary Research, Pasteur Institute of Iran 89245, Tehran, Iran; 5 Microbiology Research Center (MRC), Pasteur Institute of Iran 89245, Tehran, Iran; Assistance Publique - Hopitaux de Paris Universite Paris Saclay, Clamart, France; China Agricultural University, Beijing, China

**Keywords:** *Mycobacterium tuberculosis*, drug resistance, tuberculosis, gemifloxacin resistance, phenotypic drug susceptibility testing, QRDR-negative resistance, non-canonical resistance

## Abstract

Tuberculosis (TB) remains a global health threat, with multidrug-resistant TB (MDR-TB) presenting major therapeutic challenges. Fluoroquinolones (FQs) are key second-line drugs. Resistance is typically linked to mutations within quinolone resistance-determining regions (QRDRs), though non-canonical mechanisms are increasingly recognized. To evaluate phenotypic susceptibility to gemifloxacin in clinical *Mycobacterium tuberculosis* isolates and determine the association with mutations in *gyrA* and *gyrB* QRDRs. Sixty clinical isolates (40 drug-susceptible [DS], 20 MDR) were tested using the resazurin microtiter assay (REMA). Minimum inhibitory concentrations (MICs) ranged from 0.25 to 128 µg/mL. Resistance was defined using an epidemiological cutoff value (ECOFF) of ≥8 µg/mL. Sanger sequencing of QRDR hotspots in gyrA (codons 90, 91, 94) and gyrB (codons 500–540) was performed on all six resistant isolates and two susceptible controls. MDR isolates exhibited significantly higher median MICs compared to DS isolates (4 µg/mL vs 2 µg/mL, *P* = 0.04). Six isolates (10%) were phenotypically resistant, including two DS and four MDR strains. Given our modest sample size (*n* = 60), no mutations were detected within the sequenced QRDR segments of *gyrA* and *gyrB* in any resistant isolate or control. In this hypothesis-generating study, gemifloxacin resistance was observed in *M. tuberculosis* without mutations in the sequenced QRDR regions of *gyrA*/*gyrB*, implying alternative mechanisms, such as efflux pump activity or altered permeability. While limited by sample size, these preliminary findings challenge the reliance on molecular diagnostics targeting only QRDRs and underscore the importance of phenotypic testing for accurate detection of resistance. Larger multicenter studies are warranted to validate these observations and elucidate the underlying mechanisms.

**IMPORTANCE:**

This study provides the first evidence that *Mycobacterium tuberculosis* can develop phenotypic resistance to gemifloxacin—despite having no mutations in the canonical gyrA/gyrB quinolone resistance-determining regions (QRDRs). Among 60 clinical isolates, 10% of gemifloxacin-resistant strains were QRDR-negative. Although gemifloxacin is no longer used clinically due to toxicity, its unique structure reveals hidden, non-target-based resistance mechanisms (e.g., efflux, permeability changes) that likely also compromise current fluoroquinolones like levofloxacin and moxifloxacin. This exposes a critical blind spot: current molecular diagnostics (e.g., Xpert MTB/XDR, GenoType) may falsely label resistant strains as susceptible, risking treatment failure. We argue that phenotypic drug susceptibility testing (DST) must be mandatory—not optional—in multidrug-resistant tuberculosis (MDR-TB) programs to safeguard against this silent, undetected resistance.

## INTRODUCTION

Tuberculosis (TB), caused by *Mycobacterium tuberculosis*, remains one of the world’s most devastating infectious diseases. According to the World Health Organization (WHO) Global TB Report 2024, TB remains one of the world’s deadliest infectious diseases, causing more than 1.2 million deaths and an estimated 10.8 million incident cases in 2023 (1). Progress toward TB elimination has been disrupted by the global pandemic, which strained diagnostic and treatment services and reversed years of declining mortality (2). The emergence and spread of drug-resistant TB—particularly multidrug-resistant (MDR-TB; resistant to at least rifampicin and isoniazid) and extensively drug-resistant (XDR-TB) forms—have severely complicated clinical management (3, 4). Treatment success rates for MDR-TB remain below 68% globally, largely due to prolonged, toxic, and less effective second-line regimens (5). Fluoroquinolones (FQs), including newer agents like gemifloxacin, are cornerstone drugs in these regimens due to their potent bactericidal activity against replicating and semi-dormant bacilli (6).

FQs inhibit bacterial DNA gyrase, a type II topoisomerase composed of GyrA and GyrB subunits, encoded by *gyrA* and *gyrB* genes, respectively (7). Resistance is classically attributed to missense mutations within the quinolone resistance-determining regions (QRDRs) of these genes—particularly codons 90, 91, and 94 in *gyrA*, and codons 500–540 in *gyrB* (8). However, accumulating evidence indicates that a subset of phenotypically FQ-resistant *M. tuberculosis* isolates lack these canonical mutations (9, 10), pointing to “non-canonical” resistance pathways. These may include overexpression of efflux pumps (11, 12), alterations in cell wall permeability reducing intracellular drug accumulation (13), regulatory or epigenetic adaptations (14), or mutations in non-QRDR genomic regions affecting drug-target interaction or bacterial fitness under antibiotic pressure (15). Gemifloxacin, distinguished by a C-8 methoxy group and a unique pyrrolidine D-ring substituent, exhibits enhanced affinity for both DNA gyrase and topoisomerase IV compared to earlier-generation FQs (16, 17). Despite demonstrating potent *in vitro* activity against *M. tuberculosis* comparable to levofloxacin and moxifloxacin, gemifloxacin was excluded from TB treatment regimens due to unacceptable safety concerns. Specifically, its systemic use was discontinued following reports of dose-dependent dysglycemia (both hyperglycemia and hypoglycemia), posing significant risks in populations with high rates of diabetes or metabolic comorbidities (18, 19). Consequently, gemifloxacin was excluded from clinical trials and is not incorporated into WHO or international guidelines for drug-resistant TB, which exclusively endorse levofloxacin and moxifloxacin (20). We emphasize that this study does not advocate for the clinical use of gemifloxacin in TB treatment. However, gemifloxacin’s unique structural features and discontinued clinical use paradoxically make it an ideal research probe for investigating non-canonical resistance mechanisms. Its distinct pharmacology—particularly the bulky pyrrolidinyl substituent that may interact differently with mutant or wild-type gyrase—allows us to detect resistance patterns that might be obscured when studying clinically used FQs, where selective pressure from widespread use could confound results (21, 22). Consequently, the molecular basis of gemifloxacin resistance has been almost entirely neglected, despite its unique pharmacology making it an ideal probe for detecting non-canonical resistance mechanisms that may be shared with currently used FQs. This knowledge gap has created a critical blind spot; if resistance to gemifloxacin arises through non-QRDR mechanisms—as our data suggest—these same mechanisms may also contribute to reduced susceptibility to levofloxacin and moxifloxacin, undermining the reliability of QRDR-only molecular diagnostics. Recent evaluations of third-generation sequencing platforms, such as nanopore-based assays, similarly show that while genotypic drug susceptibility testing (DST) can achieve high overall accuracy, performance is closely tied to how completely resistance determinants are characterized—for several drugs, incomplete knowledge translates into reduced sensitivity and heterogeneous results across studies (23).

To our knowledge, systematic characterization of gemifloxacin resistance in clinical isolates of *M. tuberculosis* has not previously been reported. In this study, we aimed to characterize the *in vitro* susceptibility of clinical *M. tuberculosis* isolates to gemifloxacin and investigate whether observed resistance correlates with mutations in the QRDRs of *gyrA* and *gyrB*. We included both drug-susceptible (DS) clinical isolates and the reference strain H37Rv for baseline comparison, alongside phenotypically resistant isolates. Our findings reveal that gemifloxacin resistance can emerge without mutations in the canonical QRDR regions—highlighting the complexity of FQ resistance and the urgent need to expand molecular diagnostics beyond traditional targets. This work contributes to a more nuanced, mechanism-aware understanding of FQ resistance in TB and underscores the urgent need to integrate phenotypic drug susceptibility testing into routine clinical decision-making for MDR-TB management, particularly when newer FQs like gemifloxacin are considered.

## MATERIALS AND METHODS

### Sample collection

In 2023, a total of 60 clinical isolates of *M. tuberculosis* were consecutively collected from adult patients (age ≥18 years) with microbiologically confirmed pulmonary TB at Masih Daneshvari Hospital in Tehran, Iran. The cohort comprised 40 DS isolates and 20 MDR isolates, the latter confirmed by prior phenotypic DST using the proportion method to be resistant to at least rifampicin and isoniazid. Briefly, this method compares growth on drug-containing media versus drug-free control media, with resistance defined when ≥1% of the bacterial population grows in the presence of critical drug concentrations (0.2 µg/mL for isoniazid and 40 µg/mL for rifampicin). This WHO-endorsed method remains a reference standard for phenotypic DST despite its lengthy turnaround time of 4–6 weeks (24, 25). Complete drug susceptibility profiles for all MDR isolates, including first-line and available second-line drug testing results, are provided in Table S5. All culture-positive pulmonary TB cases meeting the inclusion criteria during the study period were considered, and once 20 MDR isolates had been identified, 40 DS isolates from the same period were included to achieve a 2:1 DS:MDR ratio. Patient identifiers were removed to ensure anonymity. For a subset of patients, clinical information on prior FQ exposure and major comorbidities (including diabetes) was available from medical records; these data are summarized in Table S3.

### Isolate verification and culture preparation

Isolates were revived and cultured on Löwenstein–Jensen medium at 37°C for 3–4 weeks. Colonies exhibiting typical rough, buff-colored, cauliflower-like morphology were selected. Acid-fast staining (Ziehl–Neelsen) confirmed cord formation characteristic of *M. tuberculosis*. Biochemical identification included catalase (heat-labile), niacin accumulation, and nitrate reduction tests, all performed according to standard protocols (26). For experimental use, isolates were subcultured in Middlebrook 7H9 broth supplemented with 10% OADC and 0.5% glycerol, incubated at 37°C with 5% CO₂ until mid-logarithmic phase (OD₆₀₀ ~ 0.6–0.8). Growth was verified by optical density measured at 600 nm (NanoDrop One, Thermo Fisher Scientific) and confirmed by microscopic examination following Ziehl–Neelsen staining. The reference strain *M. tuberculosis* H37Rv (ATCC 27294) was included in all assays as a quality control to monitor assay performance and reproducibility. Additionally, two clinical isolates from the DS group (DS-C1 and DS-C2), exhibiting consistently low gemifloxacin minimum inhibitory concentrations (MICs) (≤0.25 µg/mL), were selected as phenotypic controls for the sequencing analysis to represent highly susceptible profiles within the study cohort.

### Phenotypic drug susceptibility testing: resazurin microtiter assay

**Fig 1:**
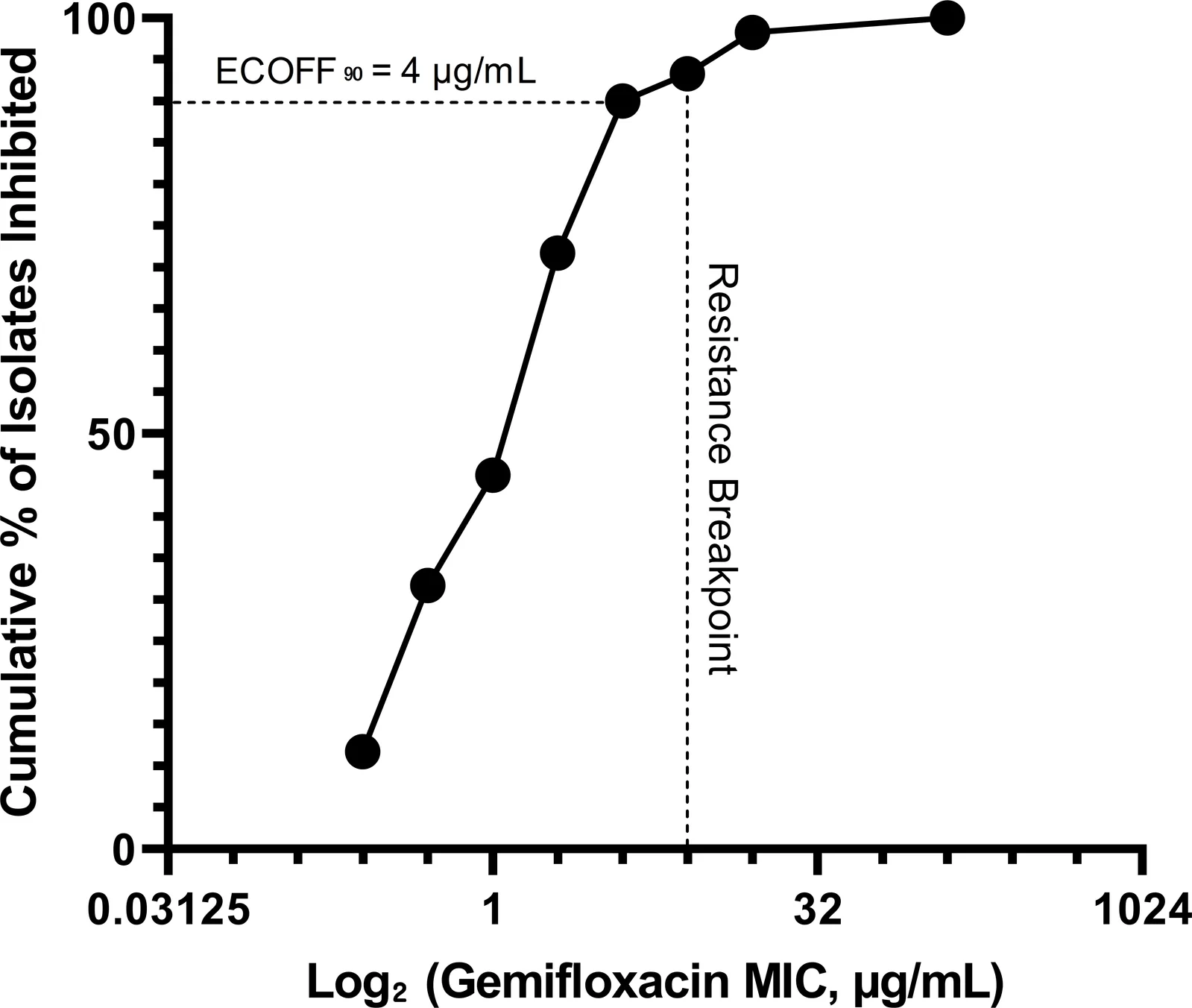
Cumulative inhibition curve for gemifloxacin against 60 clinical *M. tuberculosis* isolates (40 DS and 20 MDR). The curve shows the cumulative percentage of isolates inhibited at or below a given MIC. The dashed line at 4 µg/mL indicates the putative epidemiological cut-off (ECOFF90), corresponding to the MIC inhibiting 90% of isolates. The vertical dashed line at 8 µg/mL indicates the resistance breakpoint (isolates with MIC ≥ 8 µg/mL classified as resistant). Based on this breakpoint, 54/60 (90%) isolates were susceptible and 6/60 (10%) were resistant. Breakpoint selection was based on the bimodal distribution, pharmacokinetic considerations, and comparison with other fluoroquinolones (see Materials and Methods for detailed justification).

Gemifloxacin susceptibility was assessed using the resazurin microtiter assay (REMA) in 96-well flat-bottom plates, adhering to a methodology endorsed by the WHO, with minor adaptations (27). Initially, gemifloxacin (obtained from Sigma-Aldrich) was dissolved in sterile distilled water and serially diluted twofold across columns 2 to 11, resulting in final concentrations ranging from 0.015 to 128 µg/mL. Bacterial suspensions were prepared by adjusting them to a McFarland 1.0 standard and subsequently diluting this suspension 1:20 in 7H9-OADC broth. A volume of 100 µL of the diluted inoculum, corresponding to approximately 1 × 10^5^ CFU/mL (calculated from McFarland 1.0 standard and 1:20 dilution), was added to each well. Controls included a growth control (no drug), a sterility control (drug without bacteria), and a medium-only blank. A 7-day incubation period was selected based on prior validation studies using REMA for FQs in *M. tuberculosis* (28, 29), which demonstrated full growth inhibition by day 6. Following this incubation period, 50 µL of a 0.01% resazurin solution was added, and the plates were reincubated for an additional 24 h. The MIC was defined as the lowest concentration that inhibited 99% of growth, indicated by no color change from blue to pink. To verify reproducibility, MICs were read independently at 7 days. Because no established clinical breakpoint exists for gemifloxacin in TB, we used a distribution-based, epidemiological cut-off approach analogous to that applied for moxifloxacin (30). Examination of our MIC distribution (Fig. 1) showed that 90% of isolates were inhibited at 4 µg/mL; we therefore considered 4 µg/mL as a putative epidemiological cutoff (ECOFF90) for gemifloxacin in this collection. Given the twofold dilution series used in REMA, isolates with MIC values above this ECOFF (i.e., ≥8 µg/mL) were classified as non-wild type and designated phenotypically resistant, whereas isolates with MIC ≤ 4 µg/mL were considered within the putative wild-type distribution. This conservative definition is consistent with available pharmacokinetic/pharmacodynamic data indicating that standard gemifloxacin exposures in humans (C_max in the low µg/mL range) are unlikely to achieve effective target attainment against organisms with MICs ≥ 8 µg/mL. The distribution of gemifloxacin MICs across DS and MDR isolates, stratified by susceptibility category, is detailed in Table 1.

**TABLE 1:** Distribution of gemifloxacin MICs across DS and MDR isolates^*a*^

Isolate group	Gemifloxacin MIC (µg/mL) Number of isolates, *n* (%)								Total gemifloxacin Susceptible (≤4 µg/mL)	Total gemifloxacin Resistant (≥8 µg/mL)
	≤0.25	0.5	1	2	4	8	16	≥32		
DS (*n* = 40)	5 (12.5%)	9 (22.5%)	7 (17.5%)	12 (30.0%)	5 (12.5%)	0 (0.0%)	1 (2.5%)	1 (2.5%)	38 (95.0%)	2 (5.0%)
MDR (*n* = 20)	2 (10.0%)	3 (15.0%)	1 (5.0%)	4 (20.0%)	6 (30.0%)	2 (10.0%)	2 (10.0%)	0 (0.0%)	16 (80.0%)	4 (20.0%)
Total (*n* = 60)	7 (11.7%)	12 (20.0%)	8 (13.3%)	16 (26.7%)	11 (18.3%)	2 (3.3%)	3 (5.0%)	1 (1.7%)	54 (90.0%)	6 (10.0%)

^*a*^ Percentages for individual MICs are calculated based on the row total (*n *= 40 for DS, *n* = 20 for MDR, *n* = 60 for total). DS, drug-susceptible; MDR, multidrug-resistant.

### Genomic DNA extraction

Genomic DNA was extracted from logarithmic-phase cultures using the PREP-NA/NA DNA and RNA Extraction Kit (DNA Technology, Moscow, Russia), per manufacturer’s protocol with minor modifications: extended lysis at 65°C for 15 min, two sequential wash steps with ethanol-based buffers, and final elution in 50 µL nuclease-free water. DNA concentration and purity (A260/A280 ratio) were measured via a NanoDrop spectrophotometer (Thermo Scientific). Integrity was confirmed by 0.5% agarose gel electrophoresis (0.5× TBE, SafeStain, 80 V, 45 min).

### PCR amplification and sequencing of *gyrA* and *gyrB* QRDRs

Primers targeting QRDRs of *gyrA* (665 bp; codons 74–113) and *gyrB* (470 bp; codons 461–560) were selected and validated via Primer-BLAST against *M. tuberculosis* H37Rv (NC_000962.3). Primer sequences and amplicon coordinates are provided in Table S2. PCR was performed in 25 µL reactions using Taq 2× Master Mix (Amplicon) under the following conditions: initial denaturation at 94°C for 15 min; followed by 35 cycles of denaturation at 94°C for 30 s, annealing at 63°C (*gyrA*) or 58°C (*gyrB*) for 30 s, extension at 72°C for 30 s; and a final extension at 72°C for 10 min. Amplicons were visualized on 1% agarose gels. PCR products from all six gemifloxacin-resistant isolates and two susceptible controls (DS-C1 and DS-C2—selected for consistently low MICs ≤ 0.25 µg/mL) were purified and bidirectionally Sanger sequenced by Codon Gene Company (Tehran, Iran). Sequencing was performed using the same PCR primers for both forward and reverse reads.

### Sequence analysis

Sequences were analyzed using ChromasPro v2.1.3 and aligned to H37Rv (NC_000962.3) via BLASTn. Sequence analysis focused on the established QRDR hotspots for FQ resistance: *gyrA* codons 90, 91, and 94; and *gyrB* codons 500–540 (9).

### Statistical analysis

MIC values were log_2_-transformed prior to analysis to account for the twofold dilution series and to approximate normality, in accordance with CLSI M100 (2025) and EUCAST (2022) recommendations (31, 32). Data were analyzed using SPSS v26.0 and GraphPad Prism 9.0. Categorical variables (gemifloxacin resistance vs DS/MDR status) were compared using Fisher’s exact test (expected cell counts < 5). Differences in MIC distributions between DS and MDR isolates were assessed with the Mann–Whitney U test (Shapiro–Wilk confirmed non-normality). A cumulative inhibition curve (similar in format to a Kaplan-Meier survival curve) was generated to show the proportion of isolates inhibited at increasing concentrations. Significance threshold was determined as *P* < 0.05 (two-tailed).

## RESULTS

### Phenotypic susceptibility to gemifloxacin

Among the 60 clinical isolates tested using the REMA, the MIC values for gemifloxacin varied from 0.25 to 128 µg/mL (Table S1). The overall activity of gemifloxacin against the cohort is summarized in a cumulative inhibition curve (Fig. 1), which shows an MIC50 of 2 µg/mL and an MIC90 of 4 µg/mL. In line with an ECOFF-based approach, 4 µg/mL was taken as a putative ECOFF (ECOFF_90_), representing the upper bound of the wild-type distribution in this collection. Because MICs were determined in twofold dilutions, isolates with MIC values above this ECOFF (i.e., ≥8 µg/mL) were considered non-wild type and classified as phenotypically resistant. Using this criterion, 54 isolates (90.0%) fell within the putative wild-type distribution (MIC ≤ 4 µg/mL), while six isolates (10.0%) had MICs ≥ 8 µg/mL and were deemed resistant.

**Fig 2:**
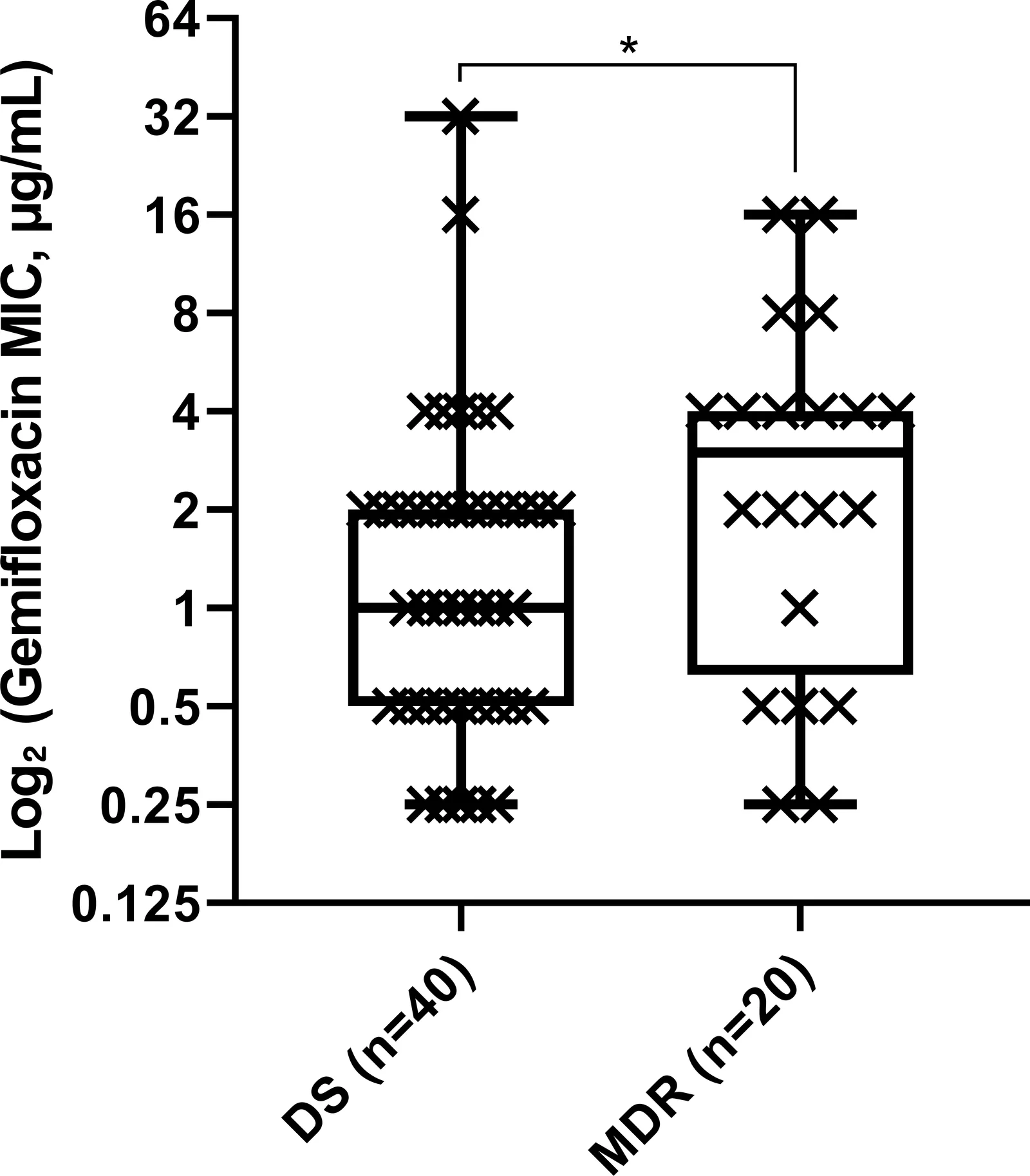
Distribution of log₂-transformed gemifloxacin MICs in drug-susceptible (DS, *n* = 40) versus multidrug-resistant (MDR, *n* = 20) *M. tuberculosis* isolates. Horizontal lines within the boxes indicate medians (DS: 1 µg/mL, interquartile range [IQR]: 0.5–2 µg/mL; MDR: 3 µg/mL, IQR: 0.625–4 µg/mL), boxes represent the IQR, and whiskers extend to the minimum and maximum values. Individual points represent the MIC for each isolate. Based on the ≥8 µg/mL breakpoint, 2/40 (5%) DS isolates and 4/20 (20%) MDR isolates were resistant. The difference in MIC distributions between groups was statistically significant (*P* = 0.04, Mann–Whitney U test). *P < 0.05.

When stratified by drug susceptibility profile, a significant difference was observed between the groups. The median MIC was 1 µg/mL (IQR: 0.5–2) in the 40 DS isolates, compared to a higher median MIC of 3 µg/mL (IQR: 0.625–4) in the 20 MDR isolates (Fig. 2; Mann-Whitney U test, *P* = 0.04). Although the rate of gemifloxacin resistance was higher in the MDR group (4/20, 20.0%) than in the DS group (2/40, 5.0%), this association did not reach statistical significance (Fisher’s exact test, *P* = 0.13). The MIC of gemifloxacin for the H37Rv reference strain was 0.25 µg/mL, consistent with previously reported susceptibility and closely resembling the values observed for clinical control isolates DS-C1 and DS-C2.

### Molecular analysis of QRDRs in resistant isolates

PCR amplification targeting the QRDRs of *gyrA* and *gyrB* was successfully performed for all six gemifloxacin-resistant isolates and two susceptible controls. Agarose gel electrophoresis confirmed that single amplicons of the expected sizes—665 bp for *gyrA* and 470 bp for *gyrB*—were generated for all isolates, with no non-specific products observed (Fig. S1). These purified PCR products were then subjected to Sanger sequencing.

All six gemifloxacin-resistant isolates, along with the two highly susceptible clinical controls (DS-C1 and DS-C2), were found to possess wild-type sequences within the sequenced QRDR regions of both *gyrA* and *gyrB*. We note that mutations outside these canonical regions or in other genes were not assessed and could potentially contribute to the observed resistance. The reference strain H37Rv was used as a quality control for all PCR and sequencing reactions, but was not part of the comparative sequencing analysis for resistance mechanisms. No nucleotide substitutions or amino acid changes were detected in the canonical resistance hotspots (codons 90, 91, and 94 of *gyrA*; codons 500–540 of *gyrB*). A manual review of the sequence chromatograms confirmed the absence of mixed base peaks or low-frequency variants that might indicate heteroresistance, with representative data providing visual confirmation of this finding presented in Fig. S2.

Analysis of the six gemifloxacin-resistant isolates revealed distinct patterns that may suggest different resistance mechanisms (Table 2; Tables S3 and S4). The two DS isolates (DS-1 and DS-2) with the highest gemifloxacin MICs (32 and 16 µg/mL, respectively) but lacking MDR status suggest potent, fluoroquinolone-specific resistance mechanisms. Among MDR isolates, MDR-1 and MDR-2 (MIC = 16 µg/mL) showed cross-resistance to levofloxacin, suggesting broad-spectrum efflux pump overexpression, while MDR-3 (MIC = 8 µg/mL) remained susceptible to other fluoroquinolones despite no prior drug exposure, indicating a highly specific, possibly novel resistance determinant.

**TABLE 2:** Characteristics of sequenced isolates^*a*^

Group	Isolate ID	Gemifloxacin MIC (µg/mL)	Resistance phenotype	*gyrA* QRDR (codons 90, 91, 94)	*gyrB* QRDR (codons 500–540)
Resistant	MDR-1	16	Resistant	Wild type	Wild type
	MDR-2	16	Resistant	Wild type	Wild type
	MDR-3	8	Resistant	Wild type	Wild type
	MDR-4	8	Resistant	Wild type	Wild type
	DS-1	32	Resistant	Wild type	Wild type
	DS-2	16	Resistant	Wild type	Wild type
Susceptible controls	DS-C1	0.25	Susceptible	Wild type	Wild type
	DS-C2	0.25	Susceptible	Wild type	Wild type

^*a*^ Isolate IDs are anonymized identifiers. Susceptible controls: DS-C1 and DS-C2 were selected as highly susceptible controls with MIC ≤ 0.25 µg/mL from the DS group.

## DISCUSSION

Our study provides robust evidence that phenotypic resistance to gemifloxacin in clinical isolates of *M. tuberculosis* can occur without detectable mutations in the canonical QRDR hotspots of *gyrA* and gyrB—a phenomenon observed in 10% of resistant isolates and absent in all susceptible controls. We included H37Rv as a reference strain for assay validation and DS-C1/DS-C2 as clinical controls to ensure that sequencing accurately distinguished wild-type susceptibility from resistance. This finding challenges the assumption that FQ resistance in TB is predominantly driven by target-site alterations and underscores the growing importance of non-canonical resistance mechanisms. Classical FQ resistance in *M. tuberculosis* is strongly associated with amino acid substitutions in GyrA (notably Ala90Val, Asp94Gly/Ala/His) and, less frequently, in GyrB (e.g., Asp500Asn, Ala543Val) (33, 34). These mutations reduce drug binding affinity by altering the enzyme’s conformational dynamics or electrostatic interface (35). However, our results align with an emerging body of literature reporting QRDR-negative FQ resistance in 5%–20% of phenotypically resistant isolates (36–38), suggesting that reliance on molecular assays targeting only *gyrA*/*gyrB* may miss a clinically significant subset of resistant cases. The structural uniqueness of gemifloxacin—characterized by a C-8 methoxy group and a bulky pyrrolidinyl substituent at the C-7 position—may partially explain this decoupling between genotype and phenotype. These modifications enhance its affinity for both DNA gyrase and topoisomerase IV in Gram-positive pathogens (39) and may alter interaction kinetics with mutant or wild-type gyrase in mycobacteria. While plausible, this hypothesis remains untested. Structural modeling of gemifloxacin–gyrase complexes in *M. tuberculosis* is currently lacking.

The cross-resistance patterns among our six resistant isolates provide clues to these alternative mechanisms. For instance, isolates MDR-1 and MDR-2 (MIC = 16 µg/mL) showed co-resistance to levofloxacin, suggesting a broad-spectrum mechanism like efflux pump overexpression. In contrast, isolate MDR-3 (MIC = 8 µg/mL) remained susceptible to other FQs, pointing toward a gemifloxacin-specific mechanism, such as a mutation affecting drug binding in a unique way. This discordant susceptibility pattern suggests that the non-canonical resistance mechanisms may not uniformly affect all FQs, possibly due to structural differences in drug-target interactions or differential substrate specificity of efflux pumps. Indeed, several non-target-based mechanisms have been proposed in the literature to explain QRDR-negative FQ resistance in *M. tuberculosis*. Overexpression of efflux pumps—particularly Rv1258c (Tap-like), Rv0194 (P55), or the Rv2686c-Rv2687c-Rv2688c tripartite system—has been linked to low-level FQ resistance in QRDR-negative isolates (12). Similarly, reduced cell wall permeability—mediated by altered mycolic acid synthesis or porin expression—can limit intracellular drug accumulation (13). Regulatory adaptations, such as upregulation of the *SigE* or *SigH* sigma factors or activation of toxin-antitoxin systems (e.g., VapC, MazF), may induce transient phenotypic tolerance that, under prolonged drug pressure, may evolve into stable resistance without target gene mutations (40–42). In our study, we did not directly assess efflux activity, permeability, or regulatory responses; thus, these mechanisms should be regarded as plausible, literature-based hypotheses rather than demonstrated causes of gemifloxacin resistance in our isolates. The inclusion of two control isolates (DS-C1 and DS-C2) with exceptionally low MICs (≤0.25 µg/mL) and wild-type QRDR sequences provided critical internal validation. Their profiles closely resemble the H37Rv reference strain, confirming assay sensitivity and sequencing fidelity. Importantly, these isolates reinforce that high *in vitro* susceptibility to gemifloxacin remains common—even among MDR strains—supporting its potential utility in salvage regimens. From a diagnostic perspective, our findings raise urgent concerns. Rapid molecular tests, such as GenoType MTBDRsl and Xpert MTB/XDR, focus almost exclusively on *gyrA* codons 88–94 and rarely include *gyrB* or non-QRDR regions. In settings where these tests guide regimen selection, QRDR-negative resistant isolates may be misclassified as susceptible—risking treatment failure, amplification of resistance, and onward transmission. Even more comprehensive sequencing-based approaches, such as Oxford Nanopore Technologies platforms, ultimately depend on current knowledge of resistance-conferring mutations. A recent systematic review and meta-analysis of nanopore sequencing for TB diagnosis and DST reported excellent overall specificity and diagnostic odds ratios for several drugs, including moxifloxacin, but also highlighted reduced accuracy for agents with incompletely characterized resistance mechanisms and substantial heterogeneity across studies (23). These findings underscore that purely genotypic assays—regardless of platform—will systematically underperform when non-canonical or unknown mechanisms are common. We therefore strongly advocate for the reinstatement of phenotypic DST (e.g., REMA, MGIT) as a mandatory adjunct to molecular diagnostics in all MDR-TB programs considering FQ-based regimens, and for the integration of broader genomic approaches (e.g., whole-genome sequencing combined with expanded mutation catalogs) to better capture QRDR-hotspot-negative resistance.

Several limitations warrant acknowledgment. First, this study is based entirely on isolates from a single tertiary care center in Tehran, Iran, with only six gemifloxacin-resistant isolates available for molecular analysis. This modest sample size limits statistical power to detect rare mutations or subgroup associations and precludes definitive conclusions about the true prevalence of QRDR-negative resistance. Importantly, the frequency of QRDR-negative FQ resistance likely varies across geographic regions due to differences in circulating *M. tuberculosis* lineages, local treatment practices, and host population genetics. Our observation that 100% (6/6) of resistant isolates lacked QRDR mutations may not be representative of global patterns and requires validation through larger, multicenter studies encompassing diverse geographic regions and at least 30–50 resistant isolates to achieve adequate statistical power. Second, we did not perform functional assays (e.g., efflux inhibition with verapamil or CCCP, RT-qPCR for pump gene expression) to confirm alternative resistance mechanisms. Our discussion of these mechanisms remains hypothetical without experimental validation. Additionally, our molecular analysis was restricted to QRDR hotspots and did not include full-length sequencing of gyrA/gyrB or whole-genome sequencing to assess other potential resistance genes. Thus, we cannot exclude the possibility of mutations outside the canonical QRDRs contributing to the observed phenotype. Future studies should integrate these approaches to identify the precise drivers of QRDR-negative resistance. Third, although we captured information on prior FQ exposure and selected comorbidities for a subset of patients (Tables S3 and S4), these data were incomplete and underpowered for robust subgroup analysis. The apparent association between prior FQ exposure and resistance (66.7% vs 32.4%) requires confirmation in larger cohorts with complete clinical data. Fourth, while REMA is WHO-endorsed and correlates well with MGIT, absolute MIC values can vary between methods. Our resistance breakpoint (≥8 µg/mL) was extrapolated from moxifloxacin ECOFFs and requires validation in larger cohorts and clinical outcome studies. Given these constraints, our findings should be considered hypothesis-generating rather than definitive. Despite these limitations, this study adds valuable evidence to the evolving paradigm of FQ resistance in TB. Although gemifloxacin is not currently recommended for TB treatment due to safety concerns (20) and is unlikely to have a routine clinical role, its potent *in vitro* activity against the majority of isolates—including MDR strains—and its unique structural properties make it a useful model compound for dissecting FQs resistance pathways that may compromise current drugs. While its systemic use is precluded by dysglycemia, its mechanism of action remains a valuable tool for understanding resistance pathways that may compromise levofloxacin and moxifloxacin. Accordingly, our findings are intended to inform mechanistic understanding and the design of improved diagnostic strategies, rather than to advocate clinical deployment of gemifloxacin.

### Conclusion

In conclusion, this hypothesis-generating study with a modest cohort (*n* = 60) reveals that phenotypic resistance to gemifloxacin in *M. tuberculosis* can emerge without detectable mutations in the classical QRDR hotspots of *gyrA* and *gyrB*. While mutations outside the sequenced regions cannot be excluded, the observation that all six resistant isolates lacked canonical QRDR mutations suggests that non-canonical resistance mechanisms warrant systematic investigation. Among 60 clinical isolates, 10% demonstrated resistance (MIC ≥ 8 µg/mL), yet Sanger sequencing of all resistant isolates—along with susceptible controls—detected no alterations in established resistance hotspots. These findings highlight the limitations of current molecular diagnostics that focus solely on gyrase mutations and emphasize the necessity of complementary phenotypic susceptibility testing to guide effective individualized therapy. While gemifloxacin retained high *in vitro* activity against the majority (90%) of isolates—including some MDR strains—its efficacy may be compromised by non-target-based resistance mechanisms, such as efflux pump overexpression, reduced membrane permeability, or regulatory adaptations. Further investigations employing transcriptomics, whole-genome sequencing, and functional assays are essential to fully characterize these pathways. Clinically, our data support the cautious inclusion of gemifloxacin in tailored regimens for MDR-TB, particularly when conventional FQs fail or are contraindicated. However, its deployment in clinical regimens must be accompanied by robust phenotypic DST or, ideally, expanded molecular diagnostics that include non-QRDR regions and efflux gene expression profiling, to avoid misclassification of QRDR-negative resistant strains. Ultimately, this work underscores the multifactorial nature of FQ resistance in TB and reinforces the need for integrated, mechanism-aware approaches to diagnosis and treatment. However, given the modest sample size and single-center design of this study, these findings should be considered preliminary and hypothesis-generating. Larger, multicenter studies with expanded genomic analysis are essential to confirm the prevalence of QRDR-negative resistance and to guide the development of comprehensive diagnostic strategies.

## Supplementary Material

Reviewer comments

## SUPPLEMENTAL MATERIAL

Supplemental figures and tables spectrum.03710-25-s0001.docx Fig. S1 and S2, and Tables S1 to S5.

OPEN PEER REVIEW reviewer-comments.pdf An accounting of the reviewer comments and feedback.

ASM does not own the copyrights to Supplemental Material that may be linked to, or accessed through, an article. The authors have granted ASM a non-exclusive, world-wide license to publish the Supplemental Material files. Please contact the corresponding author directly for reuse.
