# Supplementary material for: Gemifloxacin resistance in Mycobacterium tuberculosis without QRDR mutations in gyrA or gyrB: evidence for non-canonical resistance mechanisms
Source: Microbiol Spectr. 2026 Jun 15;14(7):e03710-25. doi: 10.1128/spectrum.03710-25 (PMC13339805; doi:10.1128/spectrum.03710-25)
Supplement: Supplemental figures and tables — Fig. S1 and S2, and Tables S1 to S5. [file spectrum.03710-25-s0001.docx]

**
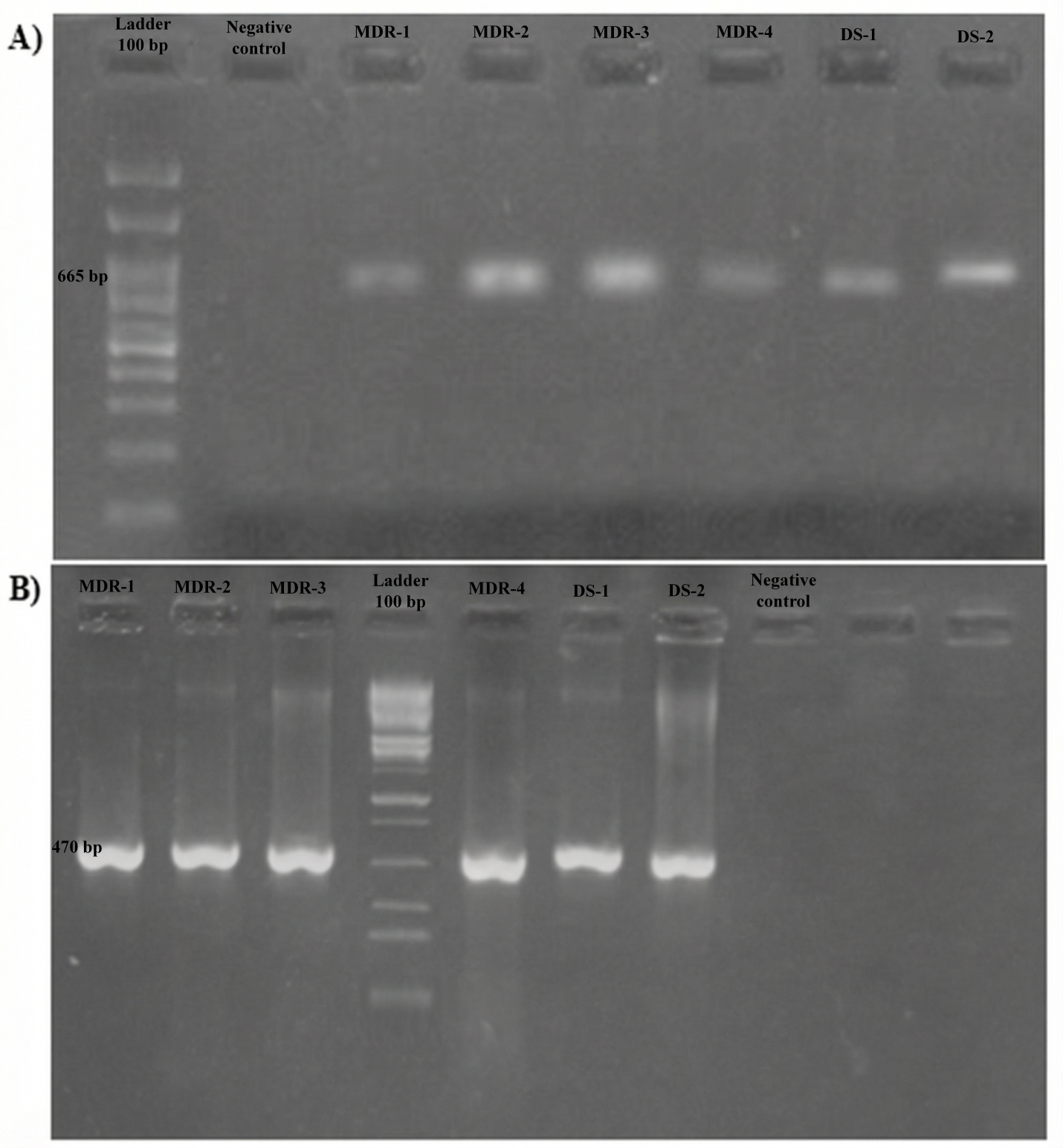
**

**Figure S1. Agarose gel analysis of PCR products for *gyrA* and *gyrB* QRDRs. A)** 100 bp DNA ladder, negative control and representative amplicons for the *gyrA* target region (expected size: 665 bp) from resistant and susceptible isolates. **B)** Representative amplicons for the *gyrB* target region (expected size: 470 bp). All PCR products were resolved on 1% agarose gel stained with SafeStain. Single, sharp bands at the expected molecular weights confirmed successful and specific amplification. Negative control lanes show no amplification, confirming absence of contamination.


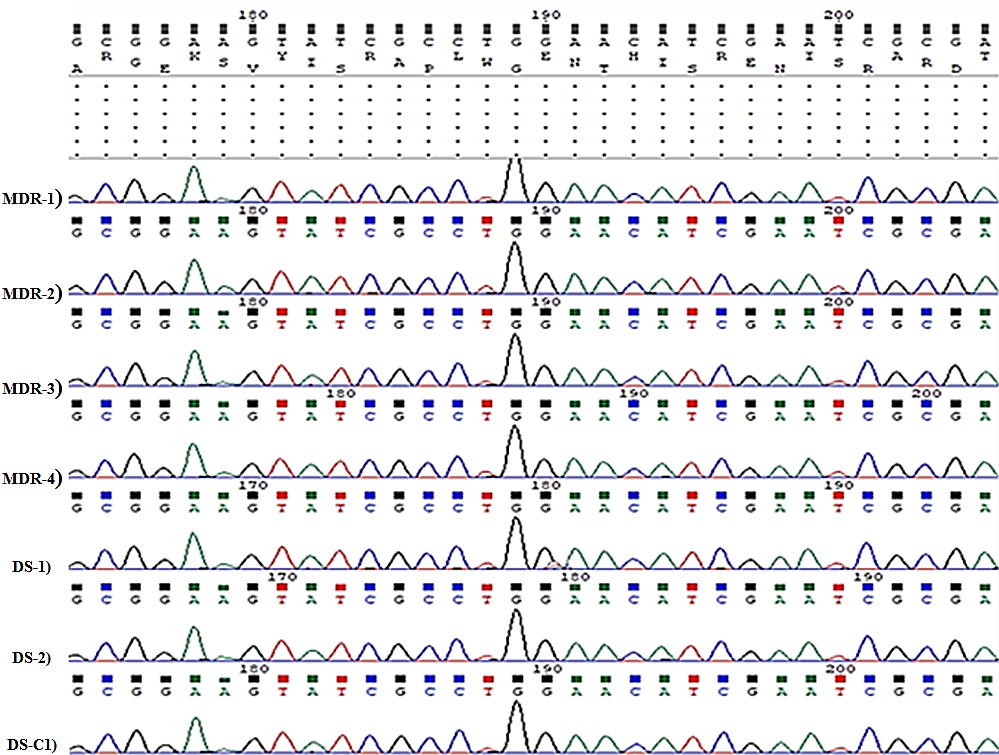


**Figure S2. Representative Sanger sequencing chromatograms confirming wild-type *gyrA* QRDR in a phenotypically resistant isolate.** The chromatogram from a gemifloxacin-resistant isolate (ID: MDR-1, MIC = 16µg/mL; MDR-2, MIC = 16µg/mL; MDR-3, MIC = 8µg/mL; MDR-4, MIC = 8µg/mL; DS-1, MIC = 32µg/mL; and DS-2, MIC = 16µg/mL) is aligned with the sequence from a highly susceptible control (ID: DS-C1, MIC = 0.25 µg/mL). All sequences show clean, unambiguous peaks with no evidence of mixed bases, insertions, or deletions, confirming wild-type status.

**Table S1. Distribution of Gemifloxacin MICs in 60 Clinical Isolates of *Mycobacterium tuberculosis***

| **MIC (µg/mL)** | **Total (n=60)** | **%** | **DS Isolates (n=40)** | **MDR Isolates (n=20)** |
| --- | --- | --- | --- | --- |
| ≤0.25 | 7 | 11.7% | 5 | 2 |
| 0.5 | 12 | 20.0% | 9 | 3 |
| 1 | 8 | 13.3% | 7 | 1 |
| 2 | 16 | 26.7% | 12 | 4 |
| 4 | 11 | 18.3% | 5 | 6 |
| 8 | 2 | 3.3% | 0 | 2 |
| 16 | 3 | 5.0% | 1 | 2 |
| ≥32 | 1 | 1.7% | 1 | 0 |
| **Resistant (≥8)** | **6** | **10.0%** | **2 (5.0%)** | **4 (20.0%)** |

*Breakpoint for resistance: ≥8 µg/mL. DS = drug-susceptible; MDR = multidrug-resistant.*

**Table S2. Primer sequences, lengths, and expected product sizes**

| **Gene** | **Sequence** | **Length (nt)** | **Expected Product Size (bp)** | **Reference (GenBank ID)** |
| --- | --- | --- | --- | --- |
| ***gyrA*** | F-TGGATGTCTAACGCAACCCT  R-TTCTCCAGCGCCCAGAACAC | 21 | 665 | [L27512.1](https://www.ncbi.nlm.nih.gov/entrez/viewer.fcgi?db=nucleotide&id=1107467) |
| ***gyrB*** | F-GTTTGAAGCCAACCCCACC  R-TGAACCGGAACAACAACGT | 20 | 470 | MK908229.1 |

**Table S3. Clinical Characteristics of Patients with Available Medical Record Data (n=43/60, 71.7%)**

| **Patient ID** | **Isolate ID** | **Group** | **Gemifloxacin MIC (µg/mL)** | **Gemifloxacin Phenotype** | **Prior FQ Exposure** | **Fluoroquinolone(s) Used*** | **TB Treatment History†** | **Diabetes** | **Other Major Comorbidities‡** |
| --- | --- | --- | --- | --- | --- | --- | --- | --- | --- |
| PT-001 | MDR-1 | MDR | 16 | R | Yes | LEV (15 d) | Retreatment | Yes | Hypertension |
| PT-002 | MDR-2 | MDR | 16 | R | Yes | Not documented | New | No | None |
| PT-003 | MDR-3 | MDR | 8 | R | No | Not documented | New | Yes | None |
| PT-004 | MDR-4 | MDR | 8 | R | Yes | Not documented | Retreatment | No | None |
| PT-005 | DS-1 | DS | 32 | R | Yes | Not documented | New | Yes | COPD |
| PT-006 | DS-2 | DS | 16 | R | No | Not documented | New | No | None |
| PT-007 | DS-C1 | DS | 0.25 | S | No | Not documented | New | No | None |
| PT-008 | DS-C2 | DS | 0.25 | S | No | Not documented | New | No | Asthma |
| PT-009 | DS-3 | DS | 2 | S | No | Not documented | New | No | Hypertension |
| PT-010 | DS-4 | DS | 1 | S | Yes | LEV (7 d, non-TB) | New | No | None |
| PT-011 | DS-5 | DS | 4 | S | No | Not documented | New | No | Malnutrition |
| PT-012 | DS-6 | DS | 2 | S | No | Not documented | New | Yes | None |
| PT-013 | DS-7 | DS | 0.5 | S | No | Not documented | New | No | None |
| PT-014 | MDR-5 | MDR | 4 | S | Yes | MOX (2 mo) | New | No | None |
| PT-015 | MDR-6 | MDR | 2 | S | Yes | LEV (2 mo) | Retreatment | Yes | Hypertension, CKD |
| PT-016 | MDR-7 | MDR | 4 | S | No | Not documented | New | No | None |
| PT-017 | MDR-8 | MDR | 1 | S | No | Not documented | New | No | None |
| PT-018 | MDR-9 | MDR | 4 | S | Yes | LEV (3 mo) | Retreatment | Yes | Diabetic retinopathy |
| PT-019 | DS-8 | DS | 0.5 | S | No | Not documented | New | No | None |
| PT-020 | DS-9 | DS | 2 | S | No | Not documented | New | No | Hepatitis B |
| PT-021 | DS-10 | DS | 1 | S | No | Not documented | New | No | None |
| PT-022 | DS-11 | DS | 4 | S | Yes | Not documented | New | Yes | None |
| PT-023 | DS-12 | DS | 2 | S | No | Not documented | New | No | Anemia |
| PT-024 | MDR-10 | MDR | 2 | S | Yes | MOX (1 mo) | New | No | None |
| PT-025 | DS-13 | DS | 0.25 | S | No | Not documented | New | No | None |
| PT-026 | DS-14 | DS | 1 | S | No | Not documented | New | No | Hypothyroidism |
| PT-027 | DS-15 | DS | 2 | S | No | Not documented | New | No | None |
| PT-028 | MDR-11 | MDR | 4 | S | Yes | LEV (2 mo) | Retreatment | No | COPD |
| PT-029 | DS-16 | DS | 0.5 | S | No | Not documented | New | No | None |
| PT-030 | DS-17 | DS | 2 | S | Yes | LEV (5 d, non-TB) | New | No | None |
| PT-031 | DS-18 | DS | 4 | S | No | Not documented | New | No | Hypertension |
| PT-032 | DS-19 | DS | 1 | S | No | Not documented | New | No | None |
| PT-033 | MDR-12 | MDR | 2 | S | No | Not documented | Retreatment | No | None |
| PT-034 | DS-20 | DS | 0.5 | S | No | Not documented | New | No | None |
| PT-035 | DS-21 | DS | 2 | S | No | Not documented | New | Yes | None |
| PT-036 | DS-22 | DS | 1 | S | Yes | CIP (7 d, non-TB) | New | No | None |
| PT-037 | MDR-13 | MDR | 4 | S | Yes | MOX (3 mo) | Retreatment | Yes | Hypertension |
| PT-038 | DS-23 | DS | 4 | S | No | Not documented | New | No | Rheumatoid arthritis |
| PT-039 | DS-24 | DS | 2 | S | No | Not documented | New | No | None |
| PT-040 | DS-25 | DS | 0.5 | S | No | Not documented | New | No | None |
| PT-041 | MDR-14 | MDR | 2 | S | No | Not documented | New | No | None |
| PT-042 | DS-26 | DS | 1 | S | No | Not documented | New | No | Depression |
| PT-043 | DS-27 | DS | 2 | S | Yes | OFX (14 d, non-TB) | New | No | None |

*LEV = levofloxacin, MOX = moxifloxacin, CIP = ciprofloxacin, OFX = ofloxacin; duration in months (mo) or days (d); "non-TB" indicates FQ prescribed for other infections †New = no prior TB treatment; Retreatment = prior TB treatment documented ‡CKD = chronic kidney disease; COPD = chronic obstructive pulmonary disease. Note: Clinical data were unavailable for 17/60 (28.3%) isolates due to incomplete medical records or referral from external facilities. Note on fluoroquinolone exposure: Among the six patients with gemifloxacin-resistant isolates, four had prior FQ exposure (66.7%): PT-001 received levofloxacin for MDR-TB treatment, while PT-002, PT-004, and PT-005 had documented FQ exposure but specific drugs were not recorded in available medical records. Two patients (PT-003 and PT-006) with resistant isolates had no documented prior FQ exposure, suggesting primary resistance or transmission of resistant strains.

**Table S4. Cross-resistance to Other Fluoroquinolones among Gemifloxacin-Resistant Isolates**

| Isolate ID | Group | Gemifloxacin MIC (µg/mL) | Levofloxacin DST* | Moxifloxacin DST* | Prior FQ Exposure† |
| --- | --- | --- | --- | --- | --- |
| MDR-1 | MDR | 16 | R | R | Yes (LEV, 1 mo) |
| MDR-2 | MDR | 16 | R | NT | Yes |
| MDR-3 | MDR | 8 | S | S | No |
| MDR-4 | MDR | 8 | R | NT | Yes |
| DS-1 | DS | 32 | NT | NT | Yes |
| DS-2 | DS | 16 | NT | NT | No |

*DST performed using proportion method on Löwenstein-Jensen medium; S = susceptible, R = resistant, NT = not tested †From Table S3 Note: Routine fluoroquinolone DST was only performed for MDR isolates as part of extended susceptibility testing protocol.

**Table S5. Drug Susceptibility Testing Profiles of MDR Isolates (n=20)**

| **Isolate ID** | **Gemifloxacin MIC (µg/mL)** | **Gemifloxacin Phenotype** | **INH** | **RIF** | **EMB** | **STR** | **PZA** | **AMK** | **KAN** | **LEV** | **MOX** |
| --- | --- | --- | --- | --- | --- | --- | --- | --- | --- | --- | --- |
| MDR-1 | 16 | R | R | R | S | R | S | S | S | R | R |
| MDR-2 | 16 | R | R | R | R | S | R | S | NT | R | NT |
| MDR-3 | 8 | R | R | R | S | S | S | S | S | S | S |
| MDR-4 | 8 | R | R | R | R | R | S | S | S | R | NT |
| MDR-5 | 4 | S | R | R | S | S | S | S | S | S | S |
| MDR-6 | 2 | S | R | R | S | R | R | S | NT | S | S |
| MDR-7 | 4 | S | R | R | R | S | S | S | S | S | S |
| MDR-8 | 1 | S | R | R | S | S | S | S | S | S | S |
| MDR-9 | 4 | S | R | R | S | R | S | S | R | S | S |
| MDR-10 | 2 | S | R | R | R | S | R | S | S | S | S |
| MDR-11 | 4 | S | R | R | S | S | S | NT | S | S | S |
| MDR-12 | 2 | S | R | R | S | S | S | S | S | S | S |
| MDR-13 | 4 | S | R | R | R | R | S | S | NT | S | S |
| MDR-14 | 2 | S | R | R | S | S | S | S | S | S | S |
| MDR-15 | 4 | S | R | R | S | R | R | S | S | NT | NT |
| MDR-16 | 2 | S | R | R | R | S | S | S | S | S | NT |
| MDR-17 | 4 | S | R | R | S | S | S | NT | NT | NT | NT |
| MDR-18 | 0.5 | S | R | R | S | S | S | S | S | S | S |
| MDR-19 | 1 | S | R | R | R | R | S | S | NT | NT | NT |
| MDR-20 | 0.5 | S | R | R | S | S | S | S | S | S | S |

INH = Isoniazid; RIF = Rifampicin; EMB = Ethambutol; STR = Streptomycin; PZA = Pyrazinamide; LEV = Levofloxacin; MOX = Moxifloxacin; AMK = Amikacin; KAN = Kanamycin; R = Resistant; S = Susceptible; NT = Not tested; All DST results except gemifloxacin were obtained using the proportion method on Löwenstein-Jensen medium according to WHO guidelines. Critical concentrations used: INH (0.2 µg/mL), RIF (40 µg/mL), EMB (2 µg/mL), STR (4 µg/mL), PZA (100 µg/mL), LEV (1.0 µg/mL), MOX (0.5 µg/mL), AMK (30 µg/mL), KAN (30 µg/mL).
