# Supplementary material for: Gemifloxacin resistance in Mycobacterium tuberculosis without QRDR mutations in gyrA or gyrB: evidence for non-canonical resistance mechanisms
Source: Microbiol Spectr. 2026 Jun 15;14(7):e03710-25. doi: 10.1128/spectrum.03710-25 (PMC13339805; doi:10.1128/spectrum.03710-25)
Supplement: Reviewer comments [file reviewer-comments.pdf]

AMERICAN SOCIETY FOR MICROBIOLOGY | Microbiology Spectrum

Gemifloxacin Resistance in Mycobacterium tuberculosis without gyrA or gyrB QRDR Mutations: Evidence for Non-Canonical Resistance Mechanisms

Niloofar Hekmatpour, Mina Hajizadeh, Shirin Yousefi, Majid Marjani, Samira Tarashi, and Seyed Davar Siadat

Corresponding Author(s): Samira Tarashi, Pasteur Institute of Iran

Review Timeline:

Submission Date:	December 3, 2025
Editorial Decision:	December 30, 2025
Revision Received:	February 10, 2026
Accepted:	February 22, 2026

Editor: Florence Doucet-Populaire

Reviewer(s): Disclosure of reviewer identity is with reference to reviewer comments included in decision letter(s). The following individuals involved in review of your submission have agreed to reveal their identity: Hao Li (Reviewer #2)

Transaction Report:

(Note: With the exception of the correction of typographical or spelling errors that could be a source of ambiguity, letters and reports are not edited. The original formatting of letters and referee reports may not be reflected in this compilation.)

DOI: https://doi.org/10.1128/spectrum.03710-25

Re: Spectrum03710-25 (Gemifloxacin Resistance in Mycobacterium tuberculosis without gyrA or gyrB QRDR Mutations: Evidence for Non-Canonical Resistance Mechanisms)

Dear Dr. Samira Tarashi:

Thank you for the privilege of reviewing your work. Below you will find my comments, instructions from the Spectrum editorial office, and the reviewer comments.

Please return the manuscript within 60 days; if you cannot complete the modification within this time period, please contact me. If you do not wish to modify the manuscript and prefer to submit it to another journal, notify me immediately so that the manuscript may be formally withdrawn from consideration by Spectrum.

Revision Guidelines
To submit your modified manuscript, log into the submission site at https://spectrum.msubmit.net/cgi-bin/main.plex. Go to Author Tasks and click the appropriate manuscript title to begin. The information you entered when you first submitted the paper will be displayed; update this as necessary. Note the following requirements:

• Upload point-by-point responses to the issues raised by the reviewers in a file named "Response to Reviewers," NOT in your cover letter.
• Upload a compare copy of the manuscript (without figures) as a "Marked-Up Manuscript" file.
• Upload a clean .DOC/.DOCX version of the revised manuscript and remove the previous version.
• Each figure must be uploaded as a separate, editable, high-resolution file (TIFF or EPS preferred), and any multipanel figures must be assembled into one file.
• Any supplemental material intended for posting by ASM should be uploaded with their legends separate from the main manuscript. You can combine all supplemental material into one file (preferred) or split it into a maximum of 10 files with all associated legends included.

For complete guidelines on revision requirements, see our Submission and Review Process webpage. Submission of a paper that does not conform to guidelines may delay acceptance of your manuscript.

Data availability: ASM policy requires that data be available to the public upon online posting of the article, so please verify all links to sequence records, if present, and make sure that each number retrieves the full record of the data. If a new accession number is not linked or a link is broken, provide Spectrum production staff with the correct URL for the record. If the accession numbers for new data are not publicly accessible before the expected online posting of the article, publication may be delayed; please contact production staff (Spectrum@asmusa.org) immediately with the expected release date.

Publication Fees: For information on publication fees and which article types are subject to charges, visit our website. If your manuscript is accepted for publication and any fees apply, you will be contacted separately about payment during the production process; please follow the instructions in that e-mail. Arrangements for payment must be made before your article is published.

ASM Membership: Corresponding authors may join or renew ASM membership to obtain discounts on publication fees. Need to upgrade your membership level? Please contact Customer Service at Service@asmusa.org.

The ASM Journals program strives for constant improvement in our submission and publication process. Please tell us how we can improve your experience by taking this quick Author Survey.

Thank you for submitting your paper to Spectrum.

Sincerely,
Florence Doucet-Populaire
Editor
Microbiology Spectrum

Reviewer #1 (Comments for the Author):

The study addresses an important and underexplored question, namely gemifloxacin resistance in Mycobacterium tuberculosis in the absence of gyrA and gyrB QRDR mutations. The overall narrative is clear, and the abstract accurately reflects the main

findings that 10 percent of isolates were gemifloxacin resistant and that none of the resistant isolates carried canonical QRDR mutations. I suggest emphasizing in the abstract and conclusion that the sample size is modest and that the findings should be interpreted as hypothesis generating.
The introduction provides good context on fluoroquinolone resistance and QRDR mutations, but the clinical positioning of gemifloxacin needs clearer framing. You explain that gemifloxacin is not currently used in WHO endorsed regimens because of safety concerns, yet later argue that it is a useful probe for non canonical resistance mechanisms. I recommend tightening this section to clearly distinguish between clinical use and its value as a research tool, and to avoid giving the impression that gemifloxacin is being advocated for routine TB treatment.
The description of isolate selection would benefit from more detail on how the 60 isolates were chosen. At present, it is not clear whether these were consecutive isolates, a convenience sample, or selected based on prior fluoroquinolone exposure. Please clarify the sampling strategy and whether any prior fluoroquinolone treatment history or comorbidities (for example diabetes) were available, since these factors could influence resistance patterns.
The REMA methodology is generally well described, but the choice of the resistance breakpoint requires stronger justification. You state that an ECOFF of at least 8 µg/mL was applied for gemifloxacin based on moxifloxacin ECOFF data, while the cumulative inhibition curve shows an ECOFF90 of 4 µg/mL in your collection. It would help to more clearly explain why 8 µg/mL was selected as the resistance cutoff, how this relates to the observed MIC distribution, and whether any pharmacokinetic or pharmacodynamic considerations were taken into account.
The statistical analysis section is partly redundant. The information about log2 transformation of MIC values and the use of non parametric tests appears twice in very similar wording. I suggest merging these sentences into a single concise description to improve readability.
The sequencing approach limited analysis to classical QRDR regions in gyrA and gyrB. While this is appropriate for a first look, the conclusion that resistance occurs "in the complete absence of gyrA or gyrB mutations" is stronger than what the data support, since non QRDR regions of these genes were not sequenced. Please soften this wording throughout the manuscript and specify that no mutations were detected within the sequenced QRDR segments, while acknowledging that mutations outside these regions or in other genes may still contribute.
The discussion of alternative resistance mechanisms is thoughtful, especially the emphasis on efflux pumps, cell wall permeability, and regulatory adaptations. However, given that no functional assays (for example efflux inhibition or expression studies) or whole genome sequencing were performed, the current wording occasionally drifts into speculation. I recommend more clearly labeling these mechanisms as hypotheses, and indicating which specific experiments would be needed in future work to test them.
Table 1 is informative but there are minor formatting issues with some of the percentages and parentheses that make the table slightly hard to read. Please carefully proofread the table to ensure that all percentages have consistent formatting and that parentheses are correctly opened and closed.
Figure 1 and Figure 2 are useful visual summaries of the MIC distribution. It would be helpful if the figure legends explicitly stated the number of isolates per group (drug susceptible versus MDR) and reiterated the resistance breakpoint used, so that readers can quickly interpret the clinical meaning of the curves and box plots without referring back to the main text.
The limitations section is appropriate but could further highlight that the study is based on isolates from a single center in Tehran and includes only six gemifloxacin resistant isolates. I suggest explicitly stating that the frequency of QRDR negative resistance may differ in other geographic regions and that larger, multicenter cohorts will be needed to validate the proportion you observed.
Please consider adding a brief comment on whether the six gemifloxacin resistant isolates showed cross resistance to other fluoroquinolones such as levofloxacin or moxifloxacin, if those data are available from routine DST. Even if these data are only available for a subset of isolates, they would help readers understand whether the non canonical mechanisms you propose are likely to affect currently used fluoroquinolones.
I recommend that you also look in to this study with DOI: 10.1038/s41598-025-90089-x. This paper synthesizes evidence on the performance of nanopore sequencing for both tuberculosis detection and drug susceptibility testing, including fluoroquinolone resistance, and highlights how unknown or non canonical resistance mechanisms can limit the accuracy of purely genotypic assays. It is directly relevant to your argument that molecular tests targeting only QRDR mutations may miss a subset of fluoroquinolone resistant isolates and supports your call for integrating phenotypic testing and broader genomic approaches into routine practice.
Finally, there are a few minor typographical and grammatical errors throughout the text, such as inconsistent spacing, repeated phrases, and occasional missing commas. A careful language edit will improve the overall professionalism of the manuscript but does not affect the scientific message.

Reviewer #2 (Comments for the Author):

In this study, the authors find that some gemifloxacin-resistant strains do not have the gyrA and gyrB mutations in the QRDR, which gives a reference for investigating the mechanisms of gemifloxacin resistance in Mtb.

Comments:
1: Line 30, the species name "Mycobacterium tuberculosis" should be in italic.
2: Line 34: This sentence needs to be rephrased.

3: The authors can use the new data from the 2025 WHO TB report and cite it as the reference.
4: The authors may briefly describe the method of DST using the proportion method or cite the references.
5: In Figure S1, the authors need to add the length of the DNA marker and revise the names of the lanes, and "Ladder", not "Lader", on Figure S1B.
6: The authors may also give the DST profiles of all the MDR strains, maybe as one supplementary table.
7: Did the authors check the MICs of the other FQs, such as levofloxacin and moxifloxacin against the isolated strains?
8: The authors may research more and find a potential mechanism for the 6 strains.
9: Did the patients enrolled in this study, especially the patients who are infected by the 6 selected strains, have the FQs treatment before? The authors may give this information.

Dear Editor and Reviewers,

We sincerely thank you for your thorough and constructive review of our manuscript entitled "Gemifloxacin Resistance in Mycobacterium tuberculosis without gyrA or gyrB QRDR Mutations: Evidence for Non-Canonical Resistance Mechanisms." We greatly appreciate the time and effort you have invested in providing detailed feedback that has significantly strengthened our work.

We have carefully considered all comments and suggestions rose by both reviewers and have revised the manuscript accordingly. The reviewers' insights have helped us improve the overall presentation of our findings. We particularly appreciate the recognition of the importance of our work in identifying non-canonical resistance mechanisms in *M. tuberculosis*, which has critical implications for current molecular diagnostic approaches.

Below, we provide point-by-point responses to each reviewer's comments, with specific details on how we have addressed their concerns in the marked-up manuscript. All changes have been highlighted in the revised version for easy identification. We believe that these revisions have substantially improved the manuscript and hope that it now meets the standards for publication. We look forward to your further feedback.

Sincerely,
Samira Tarashi
On behalf of all co-authors

Reviewer #1:

The study addresses an important and underexplored question, namely gemifloxacin resistance in *Mycobacterium tuberculosis* in the absence of gyrA and gyrB QRDR mutations. The overall narrative is clear, and the abstract accurately reflects the main findings that 10 percent of isolates were gemifloxacin resistant and that none of the resistant isolates carried canonical QRDR mutations. I suggest emphasizing in the abstract and conclusion that the sample size is modest and that the findings should be interpreted as hypothesis generating.

Response: We appreciate this suggestion. We have revised both the Abstract (lines 39-43) and the Conclusion (lines 350, 371-375) to explicitly acknowledge the modest sample size and to frame our results as exploratory and hypothesis-generating.

The introduction provides good context on fluoroquinolone resistance and QRDR mutations, but the clinical positioning of gemifloxacin needs clearer framing. You explain that gemifloxacin is not currently used in WHO endorsed regimens because of safety concerns, yet later argue that it is a useful probe for non-canonical resistance mechanisms. I recommend tightening this section to clearly distinguish between clinical use and its value as a research tool, and to avoid giving the impression that gemifloxacin is being advocated for routine TB treatment.

Response: Thank you for this important point. We agree that the distinction between gemifloxacin's discontinued clinical use and its value as a research tool was not sufficiently clear. We have restructured and tightened the relevant section of the introduction (Lines 72-94) to clearly delineate these two aspects and avoid any impression that we are advocating for its routine clinical use.

The description of isolate selection would benefit from more detail on how the 60 isolates were

chosen. At present, it is not clear whether these were consecutive isolates, a convenience sample, or selected based on prior fluoroquinolone exposure. Please clarify the sampling strategy and whether any prior fluoroquinolone treatment history or comorbidities (for example diabetes) were available, since these factors could influence resistance patterns.

Response: We thank the reviewer for highlighting the need to better describe our sampling strategy. We have revised the Methods section (lines 114-130) to clarify the sampling approach and acknowledge the limitations (lines 326-330, 336-344) regarding clinical data availability. While comprehensive clinical information was not available for all patients due to the retrospective nature of the study, we were able to retrieve key clinical data for a subset of patients from medical records, including prior fluoroquinolone exposure and major comorbidities (Supplementary Table S3).

The REMA methodology is generally well described, but the choice of the resistance breakpoint requires stronger justification. You state that an ECOFF of at least 8 µg/mL was applied for gemifloxacin based on moxifloxacin ECOFF data, while the cumulative inhibition curve shows an ECOFF90 of 4 µg/mL in your collection. It would help to more clearly explain why 8 µg/mL was selected as the resistance cutoff, how this relates to the observed MIC distribution, and whether any pharmacokinetic or pharmacodynamic considerations were taken into account.

Response: We thank the reviewer for identifying this important point and agree that our original description of the ECOFF and resistance breakpoint was unclear. We have now revised the Methods (REMA section, lines 161-171), Results (lines 211-217), and Figure 1 caption (lines 502-508) to explicitly define 4 µg/mL as the putative ECOFF (ECOFF90) in our collection, based on the observation that 90% of isolates were inhibited at this concentration; clarify that, given the two-fold dilution series, isolates with MICs above this ECOFF (≥8 µg/mL) were classified as non-wild-type/resistant, while those with MIC ≤4 µg/mL were considered within the wild-type distribution; state that this is a distribution-based, exploratory definition in the absence of clinical outcome data, and does not represent a formal clinical breakpoint; and briefly note that available pharmacokinetic/pharmacodynamic data indicate that standard gemifloxacin exposures in humans are unlikely to suppress organisms with MICs ≥8 µg/mL, supporting our choice of this conservative threshold for defining clear resistance.

The statistical analysis section is partly redundant. The information about log2 transformation of MIC values and the use of non-parametric tests appears twice in very similar wording. I suggest merging these sentences into a single concise description to improve readability.

Response: We have removed the repeated sentence and merged the description of MIC transformation and tests into a single, concise paragraph (lines 197-205).

The sequencing approach limited analysis to classical QRDR regions in gyrA and gyrB. While this is appropriate for a first look, the conclusion that resistance occurs "in the complete absence of gyrA or gyrB mutations" is stronger than what the data support, since non QRDR regions of these genes were not sequenced. Please soften this wording throughout the manuscript and specify that no mutations were detected within the sequenced QRDR segments, while acknowledging that mutations outside these regions or in other genes may still contribute.

Response: We thank the reviewer for this important clarification. We agree that our wording was too strong given that we only sequenced the QRDR regions, not the entire gyrA and gyrB genes. We have revised the manuscript throughout (Title; Abstract, lines 42; Introduction, line 90-92, 105; Results, lines 233-235; Discussion, line 256-257 and 279-281; conclusion, lines 347-351) to accurately reflect that our findings are limited to the absence of mutations in the sequenced QRDR regions and acknowledge that mutations outside these regions could contribute to resistance.

The discussion of alternative resistance mechanisms is thoughtful, especially the emphasis on efflux pumps, cell wall permeability, and regulatory adaptations. However, given that no functional assays (for example efflux inhibition or expression studies) or whole genome sequencing were performed, the current wording occasionally drifts into speculation. I recommend more clearly labeling these mechanisms as hypotheses, and indicating which specific experiments would be needed in future work to test them.

Response: We thank the reviewer for this constructive feedback. We have revised the Discussion (lines 306-308, 320-325, 333-334) of alternative mechanisms to be more clearly distinguished between observed findings and hypothetical explanations.

Table 1 is informative but there are minor formatting issues with some of the percentages and parentheses that make the table slightly hard to read. Please carefully proofread the table to ensure that all percentages have consistent formatting and that parentheses are correctly opened and closed.

Response: Thank you for pointing this out. We have carefully revised Table 1.

Figure 1 and Figure 2 are useful visual summaries of the MIC distribution. It would be helpful if the figure legends explicitly stated the number of isolates per group (drug susceptible versus MDR) and reiterated the resistance breakpoint used, so that readers can quickly interpret the clinical meaning of the curves and box plots without referring back to the main text.

Response: We have revised both figure legends to include the number of isolates per group and the resistance breakpoint, making them self-contained and easier to interpret.

The limitations section is appropriate but could further highlight that the study is based on isolates from a single center in Tehran and includes only six gemifloxacin resistant isolates. I suggest explicitly stating that the frequency of QRDR negative resistance may differ in other geographic regions and that larger, multicenter cohorts will be needed to validate the proportion you observed.

Response: We have expanded the limitations section to explicitly acknowledge the single-center design, the small number of resistant isolates, and the potential for geographic variation in resistance patterns (lines 309-318). These additions provide appropriate context for interpreting our findings.

Please consider adding a brief comment on whether the six gemifloxacin resistant isolates showed cross resistance to other fluoroquinolones such as levofloxacin or moxifloxacin, if those data are available from routine DST. Even if these data are only available for a subset of isolates, they would help readers understand whether the non-canonical mechanisms you propose are likely to affect currently used fluoroquinolones.

Response: We thank the reviewer for this important suggestion. Unfortunately, routine DST for levofloxacin and moxifloxacin was not performed on all isolates at the time of collection, as fluoroquinolone susceptibility testing is not routinely conducted for drug-susceptible isolates in our setting. However, we have retrievable DST data for the four MDR isolates that showed gemifloxacin resistance, as these underwent extended susceptibility testing. We have added this information as a new supplementary table (Table S4) and included relevant discussion (lines 326-330) in the manuscript.

I recommend that you also look in to this study with DOI: 10.1038/s41598-025-90089-x. This paper synthesizes evidence on the performance of nanopore sequencing for both tuberculosis detection and drug susceptibility testing, including fluoroquinolone resistance, and highlights how unknown or non-canonical resistance mechanisms can limit the accuracy of purely genotypic assays. It is directly relevant to your argument that molecular tests targeting only QRDR mutations may miss a subset of fluoroquinolone resistant isolates and supports your call for integrating phenotypic testing and broader genomic approaches into routine practice.
Response: We sincerely thank the reviewer for bringing this highly relevant systematic review to our attention. The Carandang et al. (2025) meta-analysis provides crucial supporting evidence for our findings and strengthens our argument about the limitations of purely genotypic approaches. We have carefully reviewed this paper and incorporated its key findings into our introduction (lines 94-68) and discussion (lines 296-303).

Finally, there are a few minor typographical and grammatical errors throughout the text, such as inconsistent spacing, repeated phrases, and occasional missing commas. A careful language edit will improve the overall professionalism of the manuscript but does not affect the scientific message.
Response: We thank the reviewer for this careful observation. The manuscript has been carefully proofread by all co-authors, with particular attention to consistency in terminology, formatting, and style. We believe these corrections have improved the clarity and professionalism of the manuscript while maintaining the scientific content intact.
We appreciate the reviewer's attention to these details, which has helped us present our findings in a more polished and professional manner.

Reviewer #2:

In this study, the authors find that some gemifloxacin-resistant strains do not have the gyrA and gyrB mutations in the QRDR, which gives a reference for investigating the mechanisms of gemifloxacin resistance in Mtb.

Comments:
1: Line 30, the species name "Mycobacterium tuberculosis" should be in italic.
Response: Thank you for pointing this out. We have corrected this instance and ensured that *Mycobacterium tuberculosis* is consistently italicized throughout the manuscript.

2: Line 34: This sentence needs to be rephrased.
Response: We thank the reviewer for identifying this awkwardly constructed sentence. We have rephrased it to improve clarity and readability.

3: The authors can use the new data from the 2025 WHO TB report and cite it as the reference.
Response: We have updated our manuscript to incorporate data from the WHO Global Tuberculosis Report 2024, which is the most recent report.

4: The authors may briefly describe the method of DST using the proportion method or cite the references.

Response: We thank the reviewer for this suggestion. We have added a brief description of the proportion method and included appropriate references to provide readers with better context for this standard DST approach (lines 118-124).

5: In Figure S1, the authors need to add the length of the DNA marker and revise the names of the lanes, and "Ladder", not "Lader", on Figure S1B.

Response: We thank the reviewer for identifying these important figure corrections. We have revised Figure S1.

6: The authors may also give the DST profiles of all the MDR strains, maybe as one supplementary table.

Response: We thank the reviewer for this valuable suggestion. We have created a new supplementary table (Table S5) presenting the complete drug susceptibility testing profiles of all 20 MDR isolates. This provides important context about the resistance patterns in our study population.

7: Did the authors check the MICs of the other FQs, such as levofloxacin and moxifloxacin against the isolated strains?

Response: We thank the reviewer for this important question. Unfortunately, we did not perform MIC testing for levofloxacin and moxifloxacin across all isolates. However, we do have phenotypic DST results using the proportion method for these fluoroquinolones for the MDR isolates, as these underwent extended susceptibility testing per our laboratory protocol. We have presented these data in Supplementary Table S4 (cross-resistance patterns) and Table S5 (complete DST profiles).

8: The authors may research more and find a potential mechanism for the 6 strains.

Response: We thank the reviewer for this important suggestion. We agree that identifying the specific mechanisms underlying QRDR-negative resistance in our six strains would significantly strengthen our findings. While comprehensive mechanistic studies were beyond the scope of this initial investigation due to resource constraints, we have expanded our discussion to provide more detailed hypotheses based on the characteristics of each resistant isolate and outline specific experiments needed for future validation.

9: Did the patients enrolled in this study, especially the patients who are infected by the 6 selected strains, have the FQs treatment before? The authors may give this information.

Response: We thank the reviewer for this important question about treatment history. We have clinical data including fluoroquinolone exposure history for 43 of 60 patients (71.7%), including all six patients with gemifloxacin-resistant isolates. This information is detailed in Supplementary Table S3. We have highlighted this finding more prominently in the revised manuscript.

Re: Spectrum03710-25R1 (Gemifloxacin Resistance in Mycobacterium tuberculosis without gyrA or gyrB QRDR Mutations: Evidence for Non-Canonical Resistance Mechanisms)

Dear Dr. Samira Tarashi:

Your manuscript has been accepted, and I am forwarding it to the ASM production staff for publication. Your paper will first be checked to make sure all elements meet the technical requirements. ASM staff will contact you if anything needs to be revised before copyediting and production can begin. Otherwise, you will be notified when your proofs are ready to be viewed.

Data Availability: ASM policy requires that data be available to the public upon online posting of the article, so please verify all links to sequence records, if present, and make sure that each number retrieves the full record of the data. If a new accession number is not linked or a link is broken, provide production staff with the correct URL for the record. If the accession numbers for new data are not publicly accessible before the expected online posting of the article, publication may be delayed; please contact ASM production staff immediately with the expected release date.

Publication Fees: For information on publication fees and which article types have charges, please visit our website. We have partnered with Copyright Clearance Center (CCC) to collect author charges. If fees apply to your paper, you will receive a message from no-reply@copyright.com with further instructions. For questions related to paying charges through RightsLink, please contact CCC at ASM_Support@copyright.com or toll free at +1-877-622-5543. CCC makes every attempt to respond to all emails within 24 hours.

ASM Membership: Corresponding authors may join or renew ASM membership to obtain discounts on publication fees. Need to upgrade your membership level? Please contact Customer Service at Service@asmusa.org.

Embargo Policy: A press release may be issued as soon as the manuscript is posted on the Spectrum Latest Articles webpage. The corresponding author will receive an email with the subject line "ASM Journals Author Services Notification" when the article is available online.

The ASM Journals program strives for constant improvement in our submission and publication process. Please tell us how we can improve your experience by taking this quick Author Survey.

Thank you for submitting your paper to Spectrum.

Sincerely,
Florence Doucet-Populaire
Editor
Microbiology Spectrum

Reviewer #2 (Comments for the Author):

1: The three line tables should be used for all the tables.
2: In the figure 2, it maybe better for different marks representing different groups.
